# Crop Rotation with Resistant Potato and *Solanum sisymbriifolium* to Control *Globodera pallida*

**DOI:** 10.2478/jofnem-2025-0054

**Published:** 2025-12-07

**Authors:** Paige Hickman, Louise-Marie Dandurand

**Affiliations:** Department of Entomology, Plant Pathology, and Nematology, University of Idaho, Moscow 83844, ID

**Keywords:** crop rotation, *Globodera pallida*, litchi tomato, pale cyst nematode, potato cyst nematode, resistance, *Solanum sisymbriifolium*, trap crop

## Abstract

*Globodera pallida*, the pale cyst nematode is a regulated pest in Idaho. This study investigated whether rotation of the trap crop *Solanum sisymbriifolium* and a resistant potato variety was effective for controlling *G. pallida* in Idaho. The highly resistant potato variety, ‘Innovator’, was used. Three-year crop rotations incorporating ‘Innovator’ and *S. sisymbriifolium* before a susceptible potato were established in an Idaho field. At the end of each growing season, the impact of each crop on the initial population used to infest soil and progeny cysts was evaluated. ‘Innovator’ and *S. sisymbriifolium* similarly reduced encysted eggs of the initial population by 70% after the first year. However, *S. sisymbriifolium* further reduced egg viability of the initial population to 58%, compared with ‘Innovator’ plots with 89% egg viability. By the end of year 3, reproduction factors (RF; final egg population/initial egg population) showed that all rotations resulted in 99%–100% overall reduction in the initial *G. pallida* population density. After 2 years of *S. sisymbriifolium*, cysts were undetectable following the susceptible potato ‘Russet Burbank’. These results demonstrate the potential of *S. sisymbriifolium* and resistant potato in rotation to reduce *G. pallida* population densities.

*Globodera pallida* (Stone) Behrens, the pale cyst nematode, is a major pest in potato worldwide. It is estimated to cause over 80% yield loss in highly infested fields (Contina et al., 2019). *G. pallida* originated from the Andes region of South America, but has spread to most major potato growing regions of the world. *G. pallida* was first found in fields in southeastern Idaho in 2006 and is regulated by USDA-APHIS (Hafez et al., 2007; Skantar et al., 2007; USDA-APHIS, 2009). The regulated area of infested and associated fields encompasses 2,611 ha in Bonneville and Bingham counties, consisting of about 1% of Idaho potato production acreage (USDA-APHIS, 2024). Idaho is the leading U.S. producer of potato, producing about 6,870,255 MT worth US$1.26 billion in 2024 (USDA-NASS, 2025a, 2025b). Potato cannot be planted in *G. pallida* infested acreage until regulatory steps have been satisfied (USDA-APHIS, 2024). Even with a low percentage of potato acreage out of production from *G. pallida* infestation, the Idaho potato industry loses US$30 million annually due to *G. pallida*, offset by only US$5 million from production of other crops (Koirala et al., 2020).

The primary control measures for *G. pallida* eradication in Idaho are stringent phytosanitary measures and fumigation with 1,3-dichloropropene (USDA-APHIS, 2024). Other control strategies such as trap crops and crop rotation may be of use to support *G. pallida* eradication efforts. Crop rotation is a common strategy for controlling pests and diseases in agricultural crops because growers can rotate to non-hosts, fallow, or resistant varieties to disrupt the pest lifecycle in an effort to reduce the pest population below an economically damaging level. Effective crop rotation is determined by several factors, including the target pest's lifecycle and host range, length of rotation, availability of resistant varieties and non-hosts, and the production feasibility and market for the rotational crop. Because *G. pallida* is highly specialized with a narrow host range, rotation to a non-host could be expected to be effective in reducing population densities. However, *G. pallida* requires specific hatching factors to hatch and can survive in a dormant state in the absence of a host for decades (Turner, 1996; Masler and Perry, 2018). Thus, rotations to a non-host or fallow are ineffective in controlling this nematode.

*Globodera pallida* persists in soil as protective cysts containing hundreds of eggs. The eggs contain second-stage juveniles (J2) which require a chemical hatching factor, typically produced by its primary host, potato, or other closely related solanaceous species (Masler and Perry, 2018; Ngala et al., 2021). Rotation to fallow or a non-host does not stimulate hatch of *G. pallida* and only results in about 20%–40% annual decline in the population due to natural attrition resulting from environmental conditions, microbes, and some spontaneous hatch (Storey, 1984; Brodie, 1996; Turner, 1996; Atkinson et al., 2001; López-Lima et al., 2013). Dormant, unhatched juveniles from field populations also begin to lose their lipid reserves, which reduces their ability to infect, but a 50% loss in lipid reserves can take at least 7.5 years (Storey, 1984; Atkinson et al., 2001). Efficient crop rotation to control *G. pallida* would incorporate resistant potato varieties or trap crops that induce hatch but prevent or limit reproduction of the nematode to decrease overall population densities.

Various crop rotation strategies have been investigated for *Globodera* spp. in different regions of the world. The tobacco cyst nematode, *Globodera tabacum tabacum* (Lownsbery & Lownsbery) is only reduced by 30%–40% after a non-host crop, but a resistant tobacco variety or tomato trap crop was found to reduce nematode population densities by as much as 96% (LaMondia, 1996). In the U.K., Whitehead et al. (1980) saw the golden nematode, *Globodera rostochiensis* (Wollenweber) Behrens controlled in rotation with nematicide before susceptible or resistant potato followed by 2 years of non-host. Tiilikkala (1991) found that in Finland, resistant potato crops reduced *G. rostochiensis* populations by 70%–80% each year, while 3 years of susceptible potato monoculture increased the population from 0.1 larvae/g soil to 265 larvae/g soil. In Canada, Bélair et al. (2016) observed that 3 years of a resistant potato reduced *G. rostochiensis* viable eggs to zero in some plots with an overall population density reduction of 95%. Additionally, 1 year of resistant potato reduced *G. rostochiensis* populations to 73%–86%, while *Solanum sisymbriifolium* Lam. as a trap crop reduced populations to 59%–66%, compared with only 28%–35% reduction by natural attrition during a non-host crop (Bélair et al., 2016).

Reduction of *G. pallida* and *G. rostochiensis* populations is limited with short rotations to non-host or fallow (Storey, 1984; Brodie, 1996; Turner, 1996; Atkinson et al., 2001; López-Lima et al., 2013). This was also demonstrated by López-Lima et al. (2013), who found that the *G. rostochiensis* infestation level only decreased by 30% in a rotation to either fallow or non-host crops, pea (*Pisum sativum* L.) and faba bean (*Vicia faba* L.). Long rotations to non-host or fallow are required to reduce the population densities below damaging levels. In the U.K., Whitehead (1995) observed that after 4 years of non-host barley, *G. pallida* and *G. rostochiensis* egg densities had decreased by as much as 86% and 71%, respectively. Long rotations of non-host barley for 4–6 years were found to reduce *G. rostochiensis* populations from 20 eggs/g soil to about 0.6–1.6 eggs/g soil, which prevented yield loss in subsequent susceptible potato (Whitehead et al., 1991). However, despite these years of a non-host crop, planting susceptible potato caused up to a 50-fold increase in the population (Whitehead et al., 1991). Regardless of natural attrition during several seasons of fallow or non-hosts, there can be a rapid resurgence of the population when susceptible potato is planted again, because some viable eggs survive season to season (Brodie, 1996; Turner, 1996; Devine et al., 1999; Ryan and Devine, 2005). Increase of *G. pallida* on susceptible potato depends on the potato variety and initial population density, but can be dramatic even in just one season (Whitehead et al., 1987, 1991). Ultimately, long rotations of non-hosts would not be practical for Idaho where rotations are typically 3 years and the goal is to eradicate *G. pallida*.

Susceptible potato has been investigated as a trap crop in rotation, but is not practical as it must be terminated before the nematode can reproduce, at the expense of the potato yield (Whitehead, 1977, 1992; Scholte, 2000a; Bélair et al., 2016). Susceptible potato was found to reduce *G. pallida* populations up to 93% when terminated after 8 weeks of growth (Whitehead, 1992). In long-term field studies, Scholte (2000a) saw that a susceptible or resistant potato trap crop grown for 8 weeks, then rotated to resistant potato or fallow, caused 92%–97% reduction in *G. pallida* populations. Compared with a season of susceptible potato, they also saw a 95%–97% reduction in *G. pallida* population density when a potato trap crop was planted before rotating to a susceptible potato (Scholte, 2000a). However, with a resistant potato alone, Halford et al. (1999) saw a 95% reduction in *G. pallida* populations while achieving a profitable yield.

In New York, *G. rostochiensis* is controlled with a crop rotation that incorporates resistant potato. Potato varieties with the *H1* resistance gene provide resistance to pathotype Ro1, the primary pathotype of the New York population (Brodie and Mai, 1989; Limantseva et al., 2014; Whitworth et al., 2018). Numerous studies were performed to develop the most effective rotation to control *G. rostochiensis* in New York. LaMondia and Brodie (1986b) saw that a resistant potato grown for 4 weeks reduced *G. rostochiensis* to 50%–60% versus a 15%–30% reduction when infested fields remained fallow. Depending on the initial population density, rotation to a season of a completely resistant potato variety can reduce population densities to 75%–95% (LaMondia and Brodie, 1986a; Brodie, 1996). In comparison, a susceptible potato can increase *G. rostochiensis* densities by as much as 25- to 50-fold (Jones, 1970; LaMondia and Brodie, 1986a). However, when *G. rostochiensis* population densities were very low at an initial population of 0.01 eggs/cm^3^ soil or less, Brodie (1996) reported no detectible population increase on a susceptible potato variety (Jones, 1970; Brodie, 1996). Studies also showed that the spread of *G. rostochiensis* is limited at populations of below 0.2 eggs/cm^3^ soil or less (Brodie, 1996). *G. rostochiensis* was therefore found to be suppressed below 0.2 eggs/cm^3^ soil by a rotation of 2 years of resistant potato, then 1 year of a non-host, followed by 1 year of susceptible potato (Brodie, 1996). Based on these research findings, this 4 years crop rotation is employed to successfully control and contain *G. rostochiensis* in New York (Brodie and Mai, 1989; Brodie, 1996; Dandurand et al., 2019b).

While there are European potato varieties with resistance to *G. pallida*, a resistant variety for commercial use in Idaho is not yet available (Whitworth et al., 2018). Most Idaho potatoes are grown for processing into french-fries, for which white-flesh oblong russet-type potatoes are preferred. Efforts to breed a *G. pallida*-resistant russet potato for Idaho are underway, so for the purposes of this study, a European variety was used to determine the effect of a resistant potato in an Idaho crop rotation to reduce *G. pallida*. This study uses ‘Innovator’, a yellow-flesh russet potato developed in the Netherlands with high resistance to *G. pallida* pathotype Pa2/3 (Buckley, 2015; Varypatakis et al., 2019), which is the same pathotype as the Idaho population (Blok and Phillips, 2012).

Another valuable tool for use in rotation to reduce *G. pallida* may be the trap crop *S. sisymbriifolium*. *S*. *sisymbriifolium* is a non-host of *G. pallida* that induces significant egg hatch, thereby dramatically reducing the population of *G. pallida* (Scholte, 2000b; Scholte and Vos, 2000; Timmermans et al., 2006). Multiple studies have found that *S. sisymbriifolium* in rotation with susceptible potato varieties reduces *G. pallida* by over 90% (Dandurand and Knudsen, 2016; Dandurand et al., 2019a; Mhatre et al., 2021). While *S. sisymbriifolium* has proven to be a successful trap crop for *G. pallida*, its use has not been widely adopted because of limited access to seed. Nevertheless, *S. sisymbriifolium* remains an effective trap crop for *G. pallida* and its efficacy in rotation with a resistant potato should be determined.

The goal of this study is to determine the potential for a resistant potato variety and the trap crop *S. sisymbriifolium* to reduce *G. pallida* populations when in rotation with susceptible potato. The impact of 3-years crop rotations on *G. pallida* were evaluated under Idaho field conditions. These rotations incorporated the resistant potato variety ‘Innovator’ and the trap crop *S. sisymbriifolium*. The rotation sequences were evaluated by assessing *G. pallida* initial population reduction and reproduction on potato after each growing season. To better understand how these crops may reduce *G. pallida* egg densities when in rotation, hatching assays were conducted in vitro to compare hatch rates caused by ‘Innovator’ and *S. sisymbriifolium.* The results from this study may contribute to development of a crop rotation plan for controlling *G. pallida* in Idaho.

## Materials and Methods

### Nematode culture

*Globodera pallida* was originally obtained from infested fields near Shelley, ID and identified with morphological and molecular methods (Skantar et al., 2007). *G. pallida* used in this study was reared on susceptible potato ‘Désirée’ or ‘Russet Burbank’ for 16 wk in the greenhouse at 18°C with 16-hr light/8-hr dark photoperiod and 60% relative humidity. Soil and roots were dried for 2 wk, then extracted with an elutriator to recover cysts (USDA-APHIS, 2009). Following extraction, cysts were dried, separated from debris, and stored at 4°C until use.

### Hatching effect of ‘Innovator’ and *S. sisymbriifolium* diffusates on *G. pallida*

The hatching effect of ‘Innovator’ and *S. sisymbriifolium* was compared in vitro with that of susceptible potatoes ‘Désirée’ and ‘Russet Burbank’, and to a bare soil-negative control using diffusate applied to *G. pallida* eggs. For diffusate production, four replicate pots per treatment were grown in randomized complete block design in the greenhouse in 15-cm-diam terracotta pots containing 1,200 g soil. Soil was a 2:1 ratio of Lane Mountain 20/30 industrial silica sand (Valley, WA) to Mission-series silt loam soil (UI-SOAC, Sandpoint, ID) that had been dried and sieved through a 5-mm mesh (Dandurand and Knudsen, 2016). Mixed sieved soil composition was 76% sand, 14% silt, and 10% clay (1% organic matter; pH 5.5). Prior to use, soil was autoclaved twice at 121°C for 90 min with 48 hr between cycles. Pots were autoclaved once at 121°C for 90 min. Greenhouse conditions were 18°C with 16-hr light to 8-hr dark photoperiod and 60% relative humidity. The potato varieties ‘Désirée’, ‘Russet Burbank’, and ‘Innovator’ were grown in tissue culture using standard media (Murashige and Skoog, 1962) for 4 wk before transplanting into the experiments. *S. sisymbriifolium* was germinated and grown for 4 wk in pro-mix potting soil (Premier Horticulture, Quakertown PA) before transplanting into pots. Two plants were planted per pot. Unplanted bare soil pots were also included as a treatment. Diffusate was collected from each pot at 4-wk and at 6-wk of growth following transplanting into soil. Diffusate from replicate pots was maintained in distinct replicates. Diffusate was collected by the soil leaching method modified from Widdowson and Wiltshire (1958) and then filtered, as described by Hickman and Dandurand (2023). The resulting diffusate samples were then frozen at −20° until use.

Hatching assays were performed within 1 mon of collecting the diffusate. The assays utilized cysts of the Idaho *G. pallida* population as described above. Cysts were first surface sterilized in 0.3% hypochlorous bleach for 5 min before being thoroughly rinsed five times in sterile deionized water (Nour et al., 2003). Cysts were then placed into 96- well plates with one cyst and 100 μl of 100 μg/ml gentamicin sulfate (VWR®, Sanborn, NY) solution per well. Cysts were hydrated for 48 hr at 18°C. Following hydration, cysts were crushed to release all eggs. Eggs, as well as second stage juveniles (J2) that were released from the egg, were counted using an inverted microscope (Leica DMi1). Diffusates were thawed to room temperature. Each diffusate replicate was applied to four cysts and 100 μl of diffusate was applied. The plates were incubated at 18°C for 2 wk and then the final hatched second stage juveniles (J2) were counted. The hatch percentage was calculated as (Final hatched J2 − Initial J2)/Initial eggs × 100. The root diffusate experiment and hatching assays were independently repeated.

### Field rotation experiment setup

**Figure 1:**
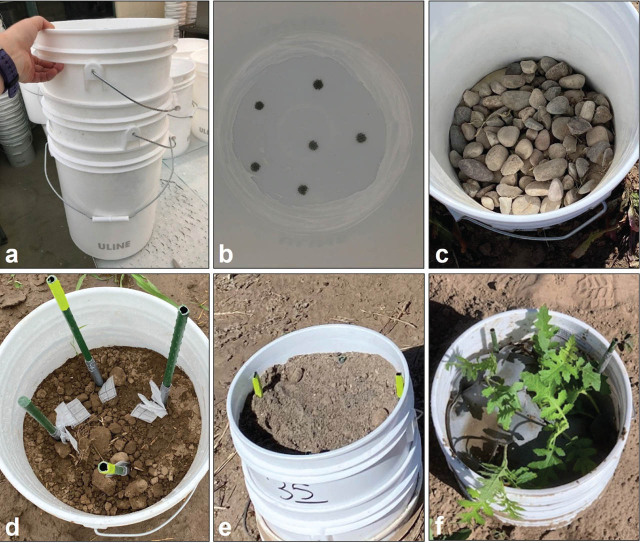
Field microplot design for *G. pallida* containment. (A) Microplot design of three 19-l buckets consisting of an upper bucket, a middle half bucket, and a lower bucket. (B) The upper bucket had six 1-cm-diam holes drilled into the bottom for drainage, covered by 250-μm pore nylon mesh caulked into the bottom of the bucket with silicone. (C) The upper bucket of the microplot was filled with a layer of river rock approximately 7.6 cm in depth. (D) *G. pallida* cyst bags were attached to stakes placed in the microplots so that cyst bags would be in the anticipated root zone at a depth of approximately 15 cm below the soil surface. (E) Field soil was added to the upper bucket, approximately 25 cm in total depth. (F) The soil surface of the upper bucket covered with a layer of landscape fabric on top of a layer of 250-μm pore nylon mesh and 4-wk-old *S. sisymbriifolium* seedlings planted into the microplot.

Two field rotation experiments were each conducted over 3 yr in microplots designed for *G. pallida* containment (Fig. 1) at a field site in a *G. pallida*-infested field in Shelley, ID. Microplot setup has been previously described by Dandurand et al. (2019a) with the following modifications. Microplots were made up of three 19-l buckets (ULINE, Pleasant Prairie, WI), consisting of an upper bucket, a middle half bucket, and a lower bucket (Fig. 1A). The upper bucket had six 1-cm-diam holes drilled into the bottom for drainage (Fig. 1B). To prevent escape of cysts, the drainage holes were covered by a nylon mesh with 250-μm pores (McMaster Carr, Elmhurst, IL) caulked into the bottom of the bucket with silicone. The lower bucket was left intact to function as containment for water that drained from the upper bucket. A third bucket was cut into half and the upper portion was used to create space between the upper bucket and the lower bucket for drainage water collection. Water or soil collected in the lower bucket was pumped out through a 10-μm pore filter bag (Pentek, Golden Valley, MN) to remove any escaped *G. pallida*. Microplots were spaced 1-m apart and placed in the ground so that the lower bucket was level with the soil surface. The upper bucket of the microplot was filled with a layer of river rock approximately 7.6 cm in depth (Fig. 1C) followed by Bock-series loam field soil (40% sand, 40% silt, 20% clay; 3.7% organic matter; pH 8.2) 25 cm in depth (Fig. 1E). As an additional *G. pallida* containment precaution, the soil surface of the upper bucket was covered with a layer of landscape fabric (Vigoro, Portland, TX) on top of a layer of nylon mesh with a 250-μm pore size (Fig. 1F). Mesh and landscape fabric were secured over the soil surface with 15-cm galvanized steel yard staples.

Microplots were inoculated with *G. pallida* cysts sealed within a nylon mesh bag with 250-μm pores approximately 5 cm × 3.8 cm and double-sealed with a table top impulse sealer (ULINE, Pleasant Prairie, WI). For easy recovery, cyst bags were tied to 0.6 m of a 22.7 kg monofilament fishing line (Zebco Holdings Inc., Tulsa, OK) with a Palomar fishing knot. The fishing line was then secured to 30-cm stakes with duct tape. The stakes were placed in the microplots so that the cyst bags would be in the anticipated root zone at a depth of approximately 15 cm below the soil surface (Fig. 1D). In the first year of rotation, microplots were inoculated at 7.5 eggs/g soil. To achieve 7.5 eggs/g soil, 300 cysts per microplot were divided into 15 cyst bags with 20 cysts per cyst bag. Cyst bags were distributed throughout the plot on five stakes with a single cyst bag and two stakes with five cyst bags. Holes were cut in the layers of mesh and landscape fabric on the microplot soil surface to allow for plant emergence and cyst bag stakes.

### Field experiment plant material and planting

*Solanum sisymbriifolium* seeds were produced at the USDA Agricultural Research Service in Prosser, WA. Seeds were germinated in pro-mix potting soil and grown for 3 wk in the greenhouse. Seedlings were acclimated outside in the shade 1 wk prior to transplantation in the field microplots. Four seedlings were transplanted per microplot. Potatoes ‘Innovator’ and ‘Russet Burbank’ were planted as mini tubers received from the University of Idaho Seed Potato Germplasm lab in Moscow, ID. Four mini tubers were planted per microplot. Barley variety ‘GemCraft’ (Hu et al., 2024) was directly seeded into the microplots at 2.5-cm planting depth. Before planting each season, the soil was tilled using a hand trowel. Osmocote Smart-Release Plant Food Plus Outdoor and Indoor slow-release fertilizer (15-9-12 NPK) (The Scotts Company, Marysville, OH) was applied on the plants at a rate of 50 g/plot. Microplots were watered daily to maintain soil moisture. Jack's Classic All Purpose 20–20–20 water soluble fertilizer (JR Peters Inc., Allentown, PA) was applied weekly at a rate of 0.5 g/l of water. Microplots were grown for 12 wk each season.

### Three-year rotation sequences

Six different rotation sequences with five replicates for each sequence arranged in randomized complete block design were grown in Idaho field conditions (Table 1). In the “Results” and “Discussion” sections, rotation crops are abbreviated and the sequences are hyphenated (Table 1). The susceptible potato ‘Russet Burbank’ is abbreviated as ‘RB’, the resistant potato ‘Innovator’ is abbreviated as ‘INN’, and *Solanum sisymbriifolium* is abbreviated as ‘*S. sisym*.’ Two trials of this experiment were performed. Rotation trial 1 was started in 2020 and was completed in 2022. Rotation trial 2 was started in 2021 and was completed in 2023. Planting took place in the beginning of June each year. Plants were grown for 12 wk following tuber sprout emergence. The growing season ended in mid-September each year. Year 1 of the rotation was the resistant potato ‘Innovator’ or *S. sisymbriifolium*. Year 2 of the rotation was ‘Innovator’, *S. sisymbriifolium*, or the non-host barley. In year 3 of the rotation, all microplots were planted with susceptible potato ‘Russet Burbank’. At the end of each growing season, plants were terminated by being cut to the soil surface and the roots were removed. Tubers from each microplot with potato were also removed. Soil and cyst bags were sampled. Microplots were dried out for 2 wk, then sealed and transported to a storage unit where they were stored until the next growing season.

**Table 1:** Rotation sequences with corresponding abbreviations.

**Year 1**	**Year 2**	**Year 3**	**Rotation sequence abbreviation^a^**
‘Innovator’ resistant potato	‘Innovator’ resistant potato	‘Russet Burbank’ susceptible potato	INN-INN-RB
‘Innovator’ resistant potato	*S. sisymbriifolium*	‘Russet Burbank’ susceptible potato	INN-*S. sisym*.-RB
‘Innovator’ resistant potato	Barley (non-host)	‘Russet Burbank’ susceptible potato	INN-Barley-RB
*S. sisymbriifolium*	‘Innovator’ resistant potato	‘Russet Burbank’ susceptible potato	*S. sisym*.-INN-RB
*S. sisymbriifolium*	*S. sisymbriifolium*	‘Russet Burbank’ susceptible potato	*S. sisym.*-*S. sisym*.-RB
*S. sisymbriifolium*	Barley (non-host)	‘Russet Burbank’ susceptible potato	*S. sisym*.-Barley-RB

a In the rotation sequences, susceptible potato ‘Russet Burbank’ is abbreviated as ‘RB’, resistant potato ‘Innovator’ is abbreviated as ‘INN’, and *Solanum sisymbriifolium* is abbreviated as ‘*S. sisym*’.

### Assessment of remaining encysted eggs

At the beginning and at the end of each growing season, one stake with a single cyst bag was removed from each microplot to sample the original *G. pallida* population used to infest soil. These cysts were used in viability and hatch assays to assess the remaining encysted eggs of the initial population used to infest the microplots. Cysts were first surface sterilized in 0.3% hypochlorous bleach for 5 min followed by five thorough rinses with sterile deionized water (Nour et al., 2003). Five cysts per replicate were used for the viability assay. Eight cysts per replicate were used for the hatching assay.

For the viability assay, cysts were placed into a 96-well plate (VWR® tissue culture plates, Radnor, PA) with one cyst and 135 μl sterile deionized water per well. Cysts were gently crushed with forceps to release the eggs. The eggs were counted using an inverted microscope (Leica DMi1). The acridine orange method was used to determine egg viability (Pillai and Dandurand, 2019). An amount of 15 μl of 100 μl/ml acridine orange stain (Life Technologies Corporation, Eugene, OR) was applied per well. Plates were incubated at 18°C in the dark for 4 hr. The stain was washed and fluorescing non-viable eggs were counted using an inverted fluorescent microscope (Leica DMi8, Leica Microsystems, Wetzlar, Germany). The percentage of viability was calculated as (viable eggs)/total eggs × 100.

Hatching assays were conducted by applying root diffusate to the remaining encysted eggs; *G. pallida* eggs for the cyst samples were collected at the beginning and end of each growing season. Four cysts per replicate were incubated with potato root diffusate (PRD) and four cysts per replicate were incubated with bare soil diffusate. Diffusate was collected from 4-wk-old ‘Désirée’ potato or bare soil by a modified soil leaching method adapted from Widdowson and Wiltshire (1958), as described above. Diffusates were kept frozen at −20°C and were less than 2 mon old at the time of use. Hatching assays were performed as previously described.

### Soil sampling to determine *G. pallida* reproduction

At the end of the first- and second-year growing seasons, 500-g soil samples were taken from each microplot. Soil sampling was done by taking five soil cores uniformly distributed throughout each plot. Soil cores were combined and homogenized, then 500 g soil was weighed out and placed into a labeled paper sample bag. The remaining soil was returned to the microplot. Soil samples were dried for 2 wk, then cysts were extracted from soil samples using the elutriator system (USDA-APHIS, 2009). After year 3 in rotation, soil and roots from each microplot were homogenized and mixed, then 2 kg of soil was sampled. The soil samples were dried for 2 wk in labeled paper sample bags. Cysts were extracted from the soil samples by elutriator (USDA-APHIS, 2009).

Following extraction, cysts were further separated from debris by acetone floatation (Brodie et al., 1976) and then counted using a dissecting microscope (Leica M80). To determine the average eggs per cyst, the eggs of five cysts per replicate were counted when sufficient cysts were available. In samples with fewer than five progeny cysts, eggs of all cysts were counted. Cysts were then crushed and the eggs were counted with an inverted microscope (Leica DMi1). The reproduction factor (RF) was calculated as *Pf*/*Pi*, where *Pi* was the starting egg population density (7.5 eggs/g soil) and *Pf* was the final egg population density (eggs/g soil).

### Data analysis

Data were analyzed using the SAS statistical package (SAS Institute Inc., Cary, NC). To determine whether data from repeated trials could be combined, analysis of variance was used to check for significant interactions between trial and treatment. If there were no interactions, data from trials were combined for further analysis. Rotation data were analyzed independently for each year. Egg count data were analyzed with a generalized linear mixed model (PROC GLIMMIX) assuming the Poisson distribution. The hatch and viability percentages were transformed by arcsin(sqrt(×/100)) before analysis to meet the assumptions of a normal distribution. To normalize the progeny cysts, *Pf*, and RF data, data were transformed by log^10^(x + 1) before analysis. The transformed data were then analyzed using a generalized linear mixed model (PROC GLIMMIX) assuming the normal distribution. Non-transformed means are shown in the tables. Means separation by least squares means was used to determine statistically significant differences between treatment means. Data were considered significantly different at *P* ≤ 0.05. Additionally, for egg hatch in PRD data to be considered significant, hatch in PRD for the treatments was compared to hatch in bare soil diffusate in a split-plot analysis of variance.

## Results

### Hatching effect of ‘Innovator’ and *S. sisymbriifolium* diffusates on *G. pallida*

**Figure 2:**
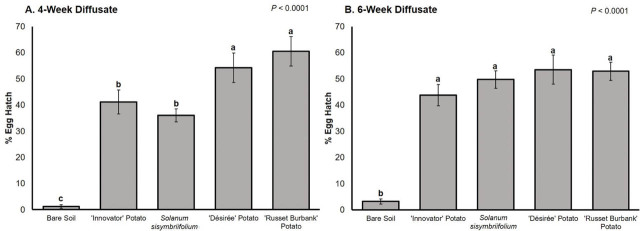
Mean percentage *G. pallida* egg hatch 2 wk after root exudate application. (A) Percentage egg hatch for root exudates was collected at 4 wk of growth. (B) Percentage of egg hatch for root exudates were collected at 6 wk of growth. Standard error of the means is indicated by the bars. Different letters indicate significantly different means based on least squares means at α = 0.05.

For the root diffusate collected at 4 wk of growth, *G. pallida* hatch was lowest in bare soil diffusate (1% hatch) and highest in susceptible potato ‘Désirée’ and ‘Russet Burbank’ diffusate (54% and 61% hatch) (Fig. 2A). The ‘Innovator’ hatching effect (41% hatch) was statistically the same as the *S. sisymbriifolium* hatching effect (36% hatch), and these were significantly lower than that of susceptible potato but significantly higher than that of bare soil (Fig. 2A). For the root diffusate collected at 6 wk of growth, hatch was again lowest in bare soil diffusate (3% hatch) (Fig. 2B). Hatch caused by root diffusate of ‘Désirée’ (54% hatch), ‘Russet Burbank’ (53% hatch), ‘Innovator’ (44% hatch), and *S. sisymbriifolium* (50% hatch) was statistically the same (Fig. 2B).

### Three-year rotation for remaining encysted eggs of initial *G. pallida* population

**Figure 3:**
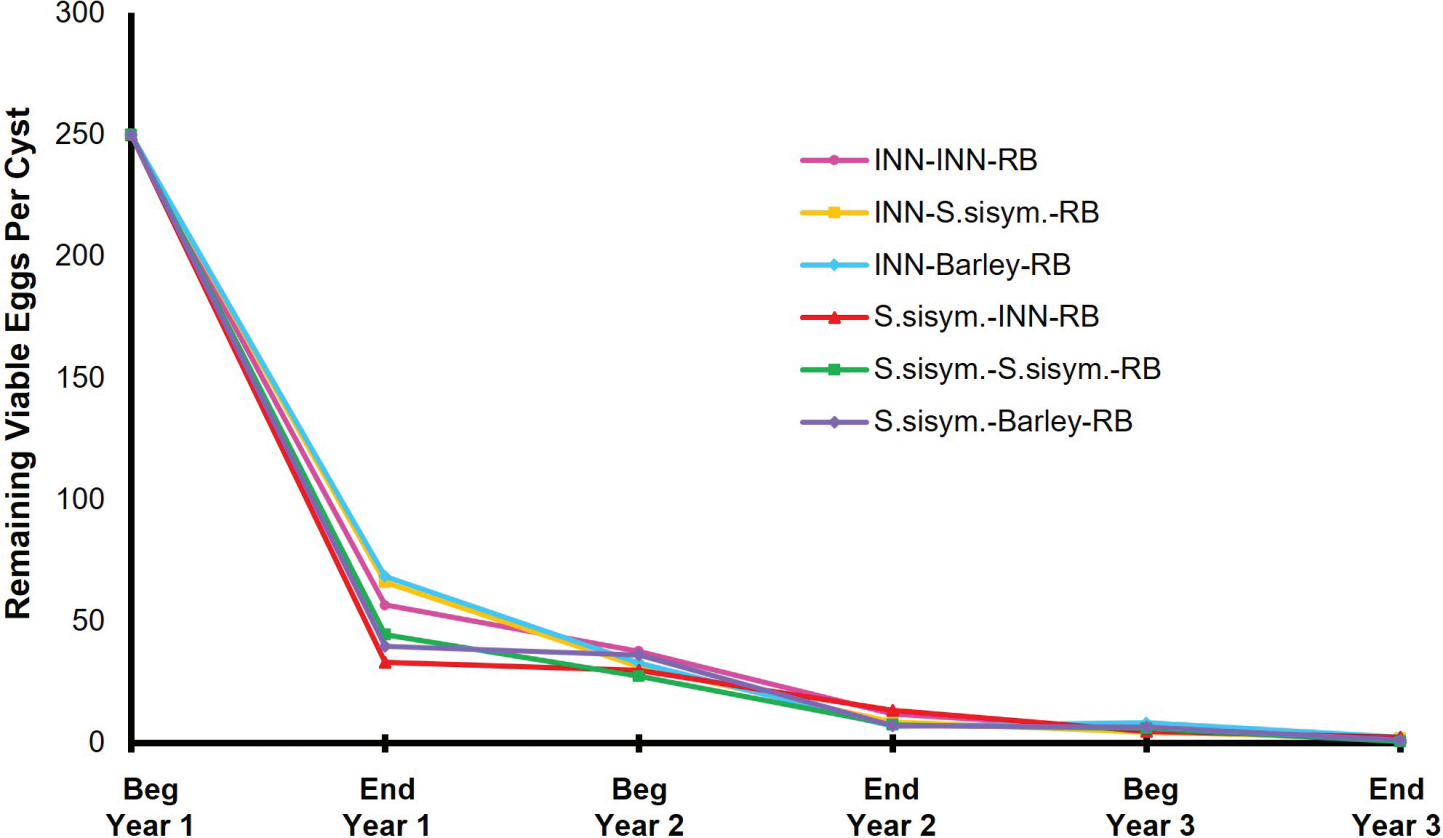
Remaining viable *G. pallida* encysted eggs per cyst of the initial *G. pallida* population over the 3-yr rotation. The initial *G. pallida* population at the beginning of year 1 in rotation started with an average of 250 eggs/cyst and an initial infestation rate of 7.5 eggs/g soil. INN, Innovator; RB, russet Burbank; *S. sisym., S. sisymbriifolium*.

Three-year rotation sequences for the six rotation plans will be referred to henceforth using hyphenated sequence abbreviations as described in the methods (Table 1). When the remaining encysted eggs of the original population were assessed at the end of the first year of rotation, the remaining *G. pallida* encysted eggs were statistically the same when ‘Innovator’ or *S. sisymbriifolium* was planted (Table 2). In the first year, ‘Innovator’ and *S. sisymbriifolium* both reduced the average remaining encysted eggs per cyst by about 70% (Fig. 3). At the end of the first season, viable eggs were 39% lower for *S. sisymbriifolium* compared with ‘Innovator’ and hatch of remaining encysted eggs was not significantly different between the two treatments (*P* < 0.001) (Table 2). The year 1 *Pf* of *S. sisymbriifolium* (*Pf* = 0.79 eggs/g soil) was significantly lower than that of ‘Innovator’ (*Pf* = 1.28 eggs/g soil) (*P* < 0.0001) (Table 3).

**Table 2:** Remaining *G. pallida* encysted egg densities, viability, and hatch at the end of the first year of rotation.^a^

**Year 1 treatment**	**Remaining encysted eggs per cyst**	**Remaining viable encysted eggs per cyst^b^**	**Egg viability (%)^b^**	**Egg hatch in PRD (%)^c^**
‘Innovator’ resistant potato	76.4 ± 5.5	63.8 ± 3.7 a	88.5 ± 2.0 a	4.2 ± 0.6
*S. sisymbriifolium*	73.0 ± 5.1	39.3 ± 2.5 b	58.4 ± 3.6 b	5.4 ± 0.7

a Data presented are means of two trials with 15 replicates per trial. Numbers following means are standard error. Significant differences are denoted by different letters in the columns based on least squares means at α = 0.05.

b Viable encysted eggs are determined through viability staining of remaining encysted eggs to reveal non-viable eggs.

c Percentage hatch in PRD is the percentage hatch of the remaining encysted eggs in PRD.

PRD, potato root diffusate.

**Table 3:** *Globodera pallida* progeny cysts, final population (*Pf*), and RF over a 3-yr rotation.^a^

**Rotation sequence^b^**	**End year 1**			**End year 2**			**End year 3**		
		
	**Progeny cysts^c^**	***Pf* (eggs/g soil)^d^**	**RF (*Pf/Pi*)^e^**	**Progeny cysts**	***Pf* (eggs/g soil)**	**RF (*Pf/Pi*)**	**Progeny cysts**	***Pf* (eggs/g soil)**	**RF (*Pf/Pi*)**
INN-INN-RB	0.1 ± 0.1	1.28 ± 0.07 a	0.26 ± 0.02 a	1.0 ± 0.2 a	0.36 ± 0.08 a	0.07 ± 0.02 a	0.5 ± 0.1 a	0.06 ± 0.01 ab	0.011 ± 0.002 ab
INN-*S. sisym*.-RB	0.1 ± 0.1	1.28 ± 0.07 a	0.26 ± 0.02 a	0.1 ± 0.1 *b*	0.17 ± 0.06 b	0.04 ± 0.01 b	0.2 ± 0.1 bc	0.04 ± 0.01 abc	0.008 ± 0.002 abc
INN-Barley-RB	0.1 ± 0.1	1.28 ± 0.07 a	0.26 ± 0.02 a	0.3 ± 0.2 b	0.16 ± 0 b	0.03 ± 0.01 b	0.4 ± 0.1 b	0.07 ± 0.02 a	0.013 ± 0.004 a
*S. sisym*.-INN-RB	0	0.79 ± 0.05 b	0.16 ± 0.01 b	0 b	0.27 ± 0.07 ab	0.05 ± 0.01 ab	0.1 ± 0 bc	0.06 ± 0.01 ab	0.010 ± 0.002 ab
*S. sisym.*-*S. sisym*.-RB	0	0.79 ± 0.05 b	0.16 ± 0.01 b	0 b	0.15 ± 0.04 b	0.03 ± 0.01 b	0 c	0.02 ± 0.01 c	0.003 ± 0.001 c
*S. sisym*.-Barley-RB	0	0.79 ± 0.05 b	0.16 ± 0.01 b	0 b	0.14 ± 0.04 b	0.03 ± 0.01 b	0.2 ± 0.1 b	0.04 ± 0.01 bc	0.007 ± 0.003 bc

a Data presented are means of two trials with five replicates per trial. Numbers following means are standard error. Significant differences are denoted by different letters in the columns based on least squares means at α = 0.05.

b In the rotation sequences, the susceptible potato ‘Russet Burbank’ is abbreviated as ‘RB’, the resistant potato ‘Innovator’ is abbreviated as ‘INN’, and *Solanum sisymbriifolium* is abbreviated as ‘*S. sisym*.’. The sequence is given as Year 1-Year 2-Year 3.

c Progeny cysts are cysts in 500-g soil sampled at the end of each growing season.

d *Pf* in eggs/g soil is the final egg population at the end of the growing season calculated by adding the remaining viable eggs of the initial population and the eggs of the progeny cysts.

e RF is reproduction factor calculated as final egg population (*Pf*) divided by initial egg population (*Pi*).

In years 2 and 3, there were no significant differences in the remaining encysted eggs, remaining viable encysted eggs, hatch, and viability of the initial *G. pallida* population for the different rotation sequences. Although there are no significant differences between the rotation sequences in years 2 and 3, the remaining viable encysted eggs of the initial population continue to decrease by about 50% season to season for all rotations (Fig. 3).

### Effect of 3-yr rotation on *G. pallida* reproduction

In general, there was low *G. pallida* reproduction for all rotation sequences. At the end of year 1, there were very low progeny cysts in ‘Innovator’ plots with 0–0.1 progeny cysts per 500-g soil (Table 3). Year 1 progeny cysts from ‘Innovator’ were not significantly different from that of *S. sisymbriifolium* (Table 3). The year 1 RF of ‘Innovator’ (RF = 0.26) was significantly greater than that of *S. sisymbriifolium* (RF = 0.16) (*P* < 0.0001), but both were <1, meaning the overall population was reduced (Table 3). In the second year, progeny cysts (*P* < 0.001) were significantly greater for the rotation sequence receiving 2 yr of ‘Innovator’ (INN-INN) compared with INN-*S. sisym*., INN-Barley, *S. sisym*.-INN, *S. sisym*.-*S. sisym*., and *S. sisym*.-Barley (Table 3). Also, at the end of year 2, *Pf* (*P* = 0.039) and RF (*P* = 0.039) were significantly greater for the rotation sequence receiving 2 yr of ‘Innovator’ (INN-INN) compared with INN-*S. sisym*., INN-Barley, *S. sisym*.-*S. sisym*., and *S. sisym*.-Barley (Table 3). At the end of year 3, the INN-INN-RB rotation produced the greatest progeny cysts (*P* < 0.0001) (Table 3). There were no detectible progeny cysts in the *S. sisym.*-*S. sisym*.-RB rotation sequence in year 3 (Table 3). By the end of year 3, the RF values indicated that the initial *G. pallida* population of 7.5 eggs/g soil was reduced by 99% to nearly 100% for all rotations (Table 3).

## Discussion

In vitro hatch assays showed that ‘Innovator’ and *S. sisymbriifolium* were alike in their effect on *G. pallida* egg hatch with significant egg hatch compared with a bare soil negative control whether 4- or 6-wk-old diffusate was used. However, the 4-wk-old diffusate hatch results showed ‘Innovator’ and *S. sisymbriifolium* had a hatching effect less than that of susceptible potato, while 6-wk-old diffusate from these two plants induced similar hatch as that of susceptible potato. This discrepancy may be explained by a variety of factors that can affect the root diffusate such as differences in plant growth, age, and health (Ochola et al., 2021). The chemical composition of root diffusate can change with the age of the plant and affect hatch (Byrne et al., 2001; Masler and Perry, 2018; Ochola et al., 2021). The hatching effect can also be impacted by concentration of the hatching factor in the diffusate, as higher concentrations can inhibit hatch (Ochola et al., 2021). Resistance genes are not known to affect root diffusate efficacy, so ‘Innovator’ resistant potato hatching effect being comparable to that of susceptible potato is an expected result (Ochola et al., 2021). In essence, the in vitro assays demonstrated that ‘Innovator’ and *S. sisymbriifolium* have the potential to cause a similar reduction in *G. pallida* egg densities through stimulating egg hatch when in rotation.

In the rotation experiment after year 1, the average remaining encysted egg densities and hatch of remaining eggs were the same for ‘Innovator’ and *S. sisymbriifolium* plots. This is likely because they caused a hatching event resulting in similar hatch percentage of the *G. pallida* population, as was shown in the in vitro hatching assays. However, viable eggs and viability percentage after year 1 were lower for *S. sisymbriifolium* than for ‘Innovator’. Non-viable eggs will not hatch during subsequent exposure to a host because their egg membrane has been disrupted (Pillai and Dandurand, 2019). *S. sisymbriifolium* may have an additional effect on egg viability. This effect may be the result of steroidal glycoalkaloids that *S. sisymbriifolium* produces (Pillai and Dandurand, 2021; Schulz et al., 2024). Pure glycoalkaloids have been found to decrease *G. pallida* hatch (Pillai and Dandurand, 2021). Schulz et al. (2024) fractionated chemicals from *S. sisymbriifolium* to try to isolate the toxic compounds and found some of the fractions to significantly reduce *G. pallida* hatch.

Most of the decline in the initial *G. pallida* population occurred in year 1 due to ‘Innovator’ and *S. sisymbriifolium*. As previous research has also shown, both of these crops are powerful tools against *G. pallida* in a crop rotation (Buckley, 2015; Dandurand and Knudsen, 2016; Dandurand et al., 2019a; Varypatakis et al., 2019; Mhatre et al., 2021). Throughout the rest of the rotation, viable encysted eggs of the initial *G. pallida* population continued to decline. This decline occurred even in rotation to the non-host barley, which should not simulate hatch of the nematode. While the barley itself may not have caused decline in *G. pallida* eggs, other factors likely contributed to natural attrition of the nematode such as degradation by microbes or spontaneous hatch (Storey, 1984; Brodie, 1996; Turner, 1996; Devine et al., 1999; Atkinson et al., 2001; López-Lima et al., 2013). Brodie (1996) found a 30%–40% decline in *G. rostochiensis* during rotation to fallow, independent of initial nematode density. Ryan and Devine (2005) reported 37% spontaneous hatch of *G. pallida* over a season of fallow. Microbial activity and spontaneous hatch have also been found to be positively correlated with soil temperature in the field (Devine et al., 1999). It is plausible that the high temperatures of southeastern Idaho, exceeding 26°C during much of the growing season, may have been a contributing factor in the decline in viable eggs in the 3-yr rotations. *G. pallida* is best adapted for temperatures between 10°C and 20°C, and temperature extremes can cause nematode mortality (Franco, 1979; Masler and Perry, 2018). Nonetheless, at the end of year 3, all rotation sequences had some remaining viable encysted eggs in the initial *G. pallida* population, which may not hatch until exposure to a host in subsequent seasons (Jones and Perry, 1978).

Reproduction of *G. pallida* was also evaluated over the 3-yr rotations. In years 1 and 2, there were a few progeny cysts recovered from ‘Innovator’ plots. ‘Innovator’ plots in rotation for 2 yr (INN-INN) had significantly higher progeny cysts and RF than the other rotations (INN-*S. sisym*., INN-Barley, *S. sisym*.-INN, *S. sisym*.-*S. sisym*., *S. sisym*.-Barley). However, the RF was much <1, indicating that the overall population of *G. pallida* was reduced when rotation included a resistant potato variety. The initial *G. pallida* population densities were reduced by all the 3-yr rotation sequences even after a susceptible potato in year 3 as evidenced by RF all being <1. Based on these results, it can be concluded that in Idaho conditions, incorporating resistant potato or *S. sisymbriifolium* in rotation before a susceptible potato is effective at reducing populations of *G. pallida*. Previous studies also support this finding as resistant potato can reduce *G. pallida* populations by 95% (Halford et al., 1999) and one crop of *S. sisymbriifolium* can reduce *G. pallida* by 90% on subsequently planted susceptible potato (Dandurand and Knudsen, 2016; Dandurand et al., 2019a; Mhatre et al., 2021). It is also important to note that no progeny cysts were recovered at the end of year 3, at 2-kg soil samples for the *S. sisym*.-*S. sisym*.-RB rotation sequence, indicating that cysts were non-detectable after a rotation sequence of 2 yr of *S. sisymbriifolium* prior to planting the susceptible variety ‘Russet Burbank’.

In conclusion, a highly resistant potato like ‘Innovator’ and the trap crop *S. sisymbriifolium* were both valuable in a crop rotation to reduce *G. pallida* population densities in Idaho. Both ‘Innovator’ and *S. sisymbriifolium* caused a hatching effect comparable to that of susceptible potato and similarly reduced *G. pallida* populations with a 70% reduction of encysted eggs in the first season. Although a highly resistant potato like ‘Innovator’ still allowed some reproduction of *G. pallida*, it resulted in an overall drastic decline in the population densities in rotation. Development of a resistant russet potato for Idaho is imperative because it could be used in rotation to control *G. pallida* and keep it below detectable levels while providing a profitable yield, as is done with *G. rostochiensis* in New York (Brodie and Mai, 1989; Brodie, 1996; Dandurand et al., 2019b). It is important to recognize that even with the use of a resistant potato or trap crop to reduce *G. pallida*, such crops must be continuously used in a rotation with susceptible potato to avoid resurgence of the population, as some viable eggs still survive season to season (Brodie, 1996; Turner, 1996; Devine et al., 1999; Ryan and Devine, 2005). Although all rotations incorporating ‘Innovator’ and *S. sisymbriifolium* before susceptible potato ‘Russet Burbank’ were shown to reduce the initial *G. pallida* population by about 99% to nearly 100%, few viable encysted eggs were still found at the end of year 3 in all plots. Ultimately, this study demonstrated that crop rotation with a highly resistant potato or the trap crop *S. sisymbriifolium* has great potential to control *G. pallida* in Idaho.

## References

[j_jofnem-2025-0054_ref_001] Atkinson H. J., Holz R. A., Riga E., Main G., Oros R., Franco J. (2001). An algorithm for optimizing rotational control of *Globodera rostochiensis* on potato crops in Bolivia. Journal of Nematology.

[j_jofnem-2025-0054_ref_002] Bélair G., Dauphinais N., Mimee B. (2016). Evaluation of cultural methods for the management of the golden nematode (*Globodera rostochiensis*) in Quebec, Canada. Canadian Journal of Plant Pathology.

[j_jofnem-2025-0054_ref_003] Blok V. C., Phillips M. S. (2012). Biological characterisation of *Globodera pallida* from Idaho. Nematology: International Journal of Fundamental and Applied Nematological Research.

[j_jofnem-2025-0054_ref_004] Brodie B. B. (1996). Effect of initial nematode density on managing *Globodera rostochiensis* with resistant cultivars and nonhosts. Journal of Nematology.

[j_jofnem-2025-0054_ref_005] Brodie B. B., Blow R. E., Brace N. L., King J. H. (1976). A new technique for handling cysts of *Heterodera rostochiensis* during routine laboratory procedures. Plant Disease Reporter.

[j_jofnem-2025-0054_ref_006] Brodie B. B., Mai W. F. (1989). Control of the golden nematode in the United States. Annual Review of Phytopathology.

[j_jofnem-2025-0054_ref_007] Buckley D. (2015). The potential of resistant cultivars to control the white potato cyst nematode *Globodera pallida*. Aspects of Applied Biology.

[j_jofnem-2025-0054_ref_008] Byrne J. T., Maher N. J., Jones P. W. (2001). Comparative responses of *Globodera rostochien-sis* and *G. pallida* to hatching chemicals. Journal of Nematology.

[j_jofnem-2025-0054_ref_009] Contina J. B., Dandurand L. M., Knudsen G. R. (2019). A predictive risk model analysis of the potato cyst nematode *Globodera pallida* in Idaho. Plant Disease.

[j_jofnem-2025-0054_ref_010] Dandurand L. M., Knudsen G. R. (2016). Effect of the trap crop *Solanum sisymbriifolium* and two biocontrol fungi on reproduction of the potato cyst nematode, *Globodera pallida*. Annals of Applied Biology.

[j_jofnem-2025-0054_ref_011] Dandurand L. M., Zasada I. A., LaMondia J. A. (2019a). Effect of the trap crop, *Solanum sisymbrii-folium*, on *Globodera pallida*, *Globodera tabacum*, and *Globodera ellingtonae*. Journal of Nematology.

[j_jofnem-2025-0054_ref_012] Dandurand L. M., Zasada I. A., Wang X., Mimee B., De Jong W., Novy R., Whitworth J., Kuhl J. C. (2019b). Current status of potato cyst nematodes in North America. Annual Review of Phytopathology.

[j_jofnem-2025-0054_ref_013] Devine K., Dunne C., O'Gara F., Jones P. (1999). The influence of in-egg mortality and spontaneous hatching on the decline of *Globodera rostochiensis* during crop rotation in the absence of the host potato crop in the field. Nematology: International Journal of Fundamental and Applied Nematological Research.

[j_jofnem-2025-0054_ref_014] Franco J. (1979). Effect of temperature on hatching and multiplication of potato cyst-nematodes. Nematologica.

[j_jofnem-2025-0054_ref_015] Hafez S. L., Sundararaj P., Handoo Z. A., Skantar A. M., Carta L. K., Chitwood D. J. (2007). First report of the pale cyst nematode, *Globodera pallida*, in the United States. Plant Disease.

[j_jofnem-2025-0054_ref_016] Halford P. D., Russell M. D., Evans K. (1999). Use of resistant and susceptible potato cultivars in the trap cropping of potato cyst nematodes, *Globodera pallida* and *G. rostochiensis*. Annals of Applied Biology.

[j_jofnem-2025-0054_ref_017] Hickman P., Dandurand L. M. (2023). Evaluation of solanaceous species as nonhost trap crops for *Globodera pallida*. Journal of Nematology.

[j_jofnem-2025-0054_ref_018] Hu G., Evans C. P., Satterfield K., Ellberg S., Marshall J. M., Schroeder K. L., Obert D. E. (2024). Registration of ‘GemCraft’ spring malting barley cultivar. Journal of Plant Registrations.

[j_jofnem-2025-0054_ref_019] Jones F. G. W. (1970). The control of the potato cyst-nematode. Journal of the Royal Society of Arts.

[j_jofnem-2025-0054_ref_020] Jones F. G. W., Perry J. N. (1978). Modelling populations of cyst-nematodes (Nematoda: Heteroderidae). Journal of Applied Ecology.

[j_jofnem-2025-0054_ref_021] Koirala S., Watson P., McIntosh C. S., Dandurand L. M. (2020). Economic impact of *Globodera pallida* on the Idaho economy. American Journal of Potato Research.

[j_jofnem-2025-0054_ref_022] LaMondia J. A. (1996). Trap crops and population management of *Globodera tabacum tabacum*. Journal of Nematology.

[j_jofnem-2025-0054_ref_023] LaMondia J. A., Brodie B. B. (1986a). Effects of initial nematode density on population dynamics of *Globodera rostochiensis* on resistant and susceptible potatoes. Journal of Nematology.

[j_jofnem-2025-0054_ref_024] LaMondia J. A., Brodie B. B. (1986b). The effect of potato trap crops and fallow on decline of *Globodera rostochiensis*. Annals of Applied Biology.

[j_jofnem-2025-0054_ref_025] Limantseva L., Mironenko N., Shuvalov O., Antonova O., Khiutti A., Novikova L., Afanasenko O., Spooner D., Gavrilenko T. (2014). Characterization of resistance to *Globodera rostochiensis* pathotype Ro1 in cultivated and wild potato species accessions from the Vavilov Institute of Plant Industry. Plant Breeding.

[j_jofnem-2025-0054_ref_026] López-Lima D., Sánchez-Nava P., Carrión G., Núñez-Sánchez A. E. (2013). 89% reduction of a potato cyst nematode population using biological control and rotation. Agronomy for Sustainable Development.

[j_jofnem-2025-0054_ref_027] Masler E. P., Perry R. N., Perry R. M., Moens M., Jones J. T. (2018). Hatch, survival and sensory perception. Cyst nematodes.

[j_jofnem-2025-0054_ref_028] Mhatre P. H., Divya K. L., Venkatasalam E. P., Bairwa A., Sudha R., Saranya C., Guru-Pirasanna-Pandid G., Sharma S. (2021). Evaluation of trap crop, *Solanum sisymbriifolium* and antagonistic crops against potato cyst nematodes, *Globodera spp*. South African Journal of Botany.

[j_jofnem-2025-0054_ref_029] Murashige T., Skoog F. (1962). A revised medium for rapid growth and bioassays with tobacco tissue cultures. Physiologia Plantarum.

[j_jofnem-2025-0054_ref_030] Ngala B., Mariette N., Ianszen M., Dewaegeneire P., Denis M. C., Porte C., Piriou C., Robilliard E., Couetil A., Nguema-Ona E., Yvin J. C., Gobert V., Beury A., Le Roux A. C., Montarry J., Fournet S. (2021). Hatching induction of cyst nematodes in bare soils drenched with root exudates under controlled conditions. Frontiers in Plant Science.

[j_jofnem-2025-0054_ref_031] Nour S. M., Lawrence J. R., Zhu H., Swerhone G. D., Welsh M., Welacky T. W., Topp E. (2003). Bacteria associated with cysts of the soybean cyst nematode (*Heterodera glycines*). Applied and Environmental Microbiology.

[j_jofnem-2025-0054_ref_032] Ochola J., Coyne D., Cortada L., Haukeland S., Ng'ang'a M., Hassanali A., Opperman C., Torto B. (2021). Cyst nematode bio-communication with plants: Implications for novel management approaches. Pest Management Science.

[j_jofnem-2025-0054_ref_033] Pillai S. S., Dandurand L. M. (2019). Evaluation of fluorescent stains for viability assessment of the potato cyst nematodes *Globodera pallida* and *G. ellingtonae*. Advances in Bioscience and Biotechnology.

[j_jofnem-2025-0054_ref_034] Pillai S. S., Dandurand L. M. (2021). Effect of steroidal glycoalkaloids on hatch and reproduction of potato cyst nematode, *Globodera pallida*. Plant Disease.

[j_jofnem-2025-0054_ref_035] Ryan A., Devine K. J. (2005). Comparison of the in-soil hatching responses of *Globodera rostochiensis* and *G. pallida* in the presence and absence of the host potato crop cv. British Queen. Nematology: International Journal of Fundamental and Applied Nematological Research.

[j_jofnem-2025-0054_ref_036] Scholte K. (2000a). Effect of potato used as a trap crop on potato cyst nematodes and other soil pathogens and on the growth of a subsequent main potato crop. Annals of Applied Biology.

[j_jofnem-2025-0054_ref_037] Scholte K. (2000b). Screening of non-tuber bearing Solanaceae for resistance to and induction of juvenile hatch of potato cyst nematodes and their potential for trap cropping. Annals of Applied Biology.

[j_jofnem-2025-0054_ref_038] Scholte K., Vos J. (2000). Effects of potential trap crops and planting date on soil infestation with potato cyst nematodes and root-knot nematodes. Annals of Applied Biology.

[j_jofnem-2025-0054_ref_039] Schulz L., Popova I., Dandurand L. M. (2024). Toxic effects of the trap crop *Solanum sisymbriifolium* on the hatch and viability of *Globodera pallida*. Journal of Nematology.

[j_jofnem-2025-0054_ref_040] Skantar A. M., Handoo Z. A., Carta L. K., Chitwood D. J. (2007). Morphological and molecular identification of *Globodera pallida* associated with potato in Idaho. Journal of Nematology.

[j_jofnem-2025-0054_ref_041] Storey R. M. J. (1984). The relationship between neutral lipid reserves and infectivity for hatched and dormant juveniles of *Globodera* spp. Annals of Applied Biology.

[j_jofnem-2025-0054_ref_042] Tiilikkala K. (1991). Effect of crop rotation on *Globodera rostochiensis* and on potato yield1. EPPO Bulletin.

[j_jofnem-2025-0054_ref_043] Timmermans B. G. H., Vos J., Stomph T. J., Van Nieuwburg J., Van der Putten P. E. L. (2006). Growth duration and root length density of *Solanum sisymbriifolium* (Lam.) as determinants of hatching of *Globodera pallida* (Stone). Annals of Applied Biology.

[j_jofnem-2025-0054_ref_044] Turner S. J. (1996). Population decline of potato cyst nematodes (*Globodera rostochiensis*, *G. pallida*) in field soils in Northern Ireland. Annals of Applied Biology.

[j_jofnem-2025-0054_ref_045] USDA-APHIS (2009). Pale potato cyst nematode national survey and diagnostic cyst sample forwarding protocols.

[j_jofnem-2025-0054_ref_046] USDA-APHIS (2024). Pale cyst nematode (PCN) eradication program, Idaho Falls, ID 2024 4^th^ quarter report (October 1-December 31).

[j_jofnem-2025-0054_ref_047] USDA-NASS (2025a). 2024 state agriculture overview: Idaho.

[j_jofnem-2025-0054_ref_048] USDA-NASS (2025b). Crop production 2024 summary.

[j_jofnem-2025-0054_ref_049] Varypatakis K., Jones J. T., Blok V. C. (2019). Susceptibility of potato varieties to populations of *Globodera pallida* selected for increased virulence. Nematology: International Journal of Fundamental and Applied Nematological Research.

[j_jofnem-2025-0054_ref_050] Whitehead A. G. (1977). Control of potato cyst-nematode, *Globodera rostochiensis* Rol, by picrolonic acid and potato trap crops. Annals of Applied Biology.

[j_jofnem-2025-0054_ref_051] Whitehead A. G. (1992). Emergence of juvenile potato cyst-nematodes *Globodera rostochiensis* and *G. pallida* and the control of *G. pallida*. Annals of Applied Biology.

[j_jofnem-2025-0054_ref_052] Whitehead A. G. (1995). Decline of potato cyst nematodes, *Globodera rostochiensis* and *G. pallida*, in spring barley microplots. Plant Pathology.

[j_jofnem-2025-0054_ref_053] Whitehead A. G., Tite D. J., Fraser J. E., French E. M. (1980). Control of potato cyst-nematode, *Globodera rostochiensis*, in a three-course rotation. The Journal of Agricultural Science.

[j_jofnem-2025-0054_ref_054] Whitehead A. G., Tite D. J., Fraser J. E., Nichols A. J. F. (1987). Effects of potato genotype, oxamyl and the numbers of potato cyst-nematodes, *Globodera rostochiensis* or *G. pallida* on tuber yields and nematode increase. Annals of Applied Biology.

[j_jofnem-2025-0054_ref_055] Whitehead A. G., Webb R. M., Beane J. (1991). Effects of rotation length and oxamyl on potato yield and the potato cyst-nematode, *Globodera rostochiensis*, in a sandy loam soil. Annals of Applied Biology.

[j_jofnem-2025-0054_ref_056] Whitworth J. L., Novy R. G., Zasada I. A., Wang X., Dandurand L. M., Kuhl J. C. (2018). Resistance of potato breeding clones and cultivars to three species of potato cyst nematode. Plant Disease.

[j_jofnem-2025-0054_ref_057] Widdowson E., Wiltshire G. H. (1958). The potato-eelworm hatching factor. Annals of Applied Biology.

